# How much do government and households spend on an episode of hospitalisation in India? A comparison for public and private hospitals in Chhattisgarh state

**DOI:** 10.1186/s13561-022-00372-0

**Published:** 2022-05-06

**Authors:** Samir Garg, Narayan Tripathi, Alok Ranjan, Kirtti Kumar Bebarta

**Affiliations:** 1 State Health Resource Centre, Raipur, Chhattisgarh India; 2 Indian Institute of Technology, Jodhpur, India; 3 State Health Resource Centre, Raipur, Chhattisgarh India

**Keywords:** Supply-side spending, Demand-side spending, Health expenditure, Universal health coverage, UHC, Provider mix, Purchasing, India, Efficiency

## Abstract

**Background:**

Improvements in the financing of healthcare services are important for developing countries like India to make progress towards universal health coverage. Inpatient-care contributes to a big share of total health expenditure in India. India has a mixed health-system with a sizeable presence of private hospitals. Existing studies show that out-of-pocket expenditure (OOPE) incurred per hospitalisation in private hospitals was greater than public facilities. But, such comparisons have not taken into account the healthcare spending by government.

**Methods:**

For a valid comparison between public and for-profit private providers, this study in Indian state of Chhattisgarh assessed the combined spending by government and households per episode of hospitalisation. The supply-side and demand-side spending from public and private sources was taken into account. The study used two datasets: a) household survey for data on hospital utilisation, OOPE, cash incentives received by patients and claims raised under publicly funded health insurance (PFHI) schemes (*n* = 903 hospitalisation episodes) b) survey of public facilities to find supply-side government spending per hospitalisation (*n* = 64 facilities).

**Results:**

Taking into account all relevant demand and supply side expenditures, the average total spending per day of hospitalisation was INR 2833 for public hospitals and INR 6788 for private hospitals. Adjusted model for logarithmic transformation of OOPE while controlling for variables including case-mix showed that a hospitalisation in private hospitals was significantly more expensive than public hospitals (coefficient = 2.9, *p* < 0.001). Hospitalisations in private hospitals were more likely to result in a PFHI claim (adjusted-odds-ratio = 1.45, *p* = 0.02) and involve a greater amount than public hospitals (coefficient = 0.27, *p* < 0.001). Propensity-score matching models confirmed the above results.

Overall, supply-side public spending contributed to 16% of total spending, demand-side spending through PFHI to 16%, cash incentives to 1% and OOPE to 67%. OOPE constituted 31% of total spending per episode in public and 86% in private hospitals.

**Conclusions:**

Government and households put together spent substantially more per hospitalisation in private hospitals than public hospitals in Chhattisgarh. This has important implications for the allocative efficiency and the desired public-private provider-mix. Using public resources for purchasing inpatient care services from private providers may not be a suitable strategy for such contexts.

**Supplementary Information:**

The online version contains supplementary material available at 10.1186/s13561-022-00372-0.

## Background

Improvements in financing of healthcare services are important for making progress towards the vision of universal health coverage (UHC) [1]. In order to draw lessons for improving financing, it is essential to understand the amount of spending on different healthcare needs of population [2]. There is a dearth of such information for low and middle income countries (LMICs) including India [2–4].

Inpatient care is an essential healthcare need and it is a bigger contributor to the total health expenditure than any other form of healthcare utilisation in India [5]. It is therefore important to know the amount of spending per episode of hospitalisation and its sources. Further, India has a mixed health system and a sizeable share of utilisation occurs in the private sector [6]. Private hospitals accounted for around 30% of inpatient episodes for the maternity care and 55% of inpatient episodes for non-maternity purposes in India [7]. The public and private sectors are financed differently and policy debates on their financing need to be informed by the comparative spending per episode of utilisation in public and private hospitals [2, 3, 8].

Most of the existing studies on spending on inpatient care in India have focused on the out of pocket expenditure (OOPE) [8–10]. These studies show that the OOPE incurred per episode in private sector was greater than in public sector [8–10]. But, such studies have not taken into account the healthcare spending by government. This makes it difficult to draw a fair comparison between private and public hospitals. Private hospitals need to earn a profit to survive whereas public hospitals have no such compulsion. This can result in greater OOPE in private hospitals as they need to charge enough to include a profit. Public hospitals receive supply-side funding from the government which private hospitals do not. A valid comparison of spending between public and private providers per episode of hospitalisation needs to be based on the combined expenditure by government and households. None of the existing studies have done that. The current study is aimed at providing a fair comparison of spending in public and private hospitals.

There exists another set of studies on unit hospitalisation expenditure in India. But, these studies have included only the supply side spending [4, 11–13]. India has implemented demand side financing in healthcare in the form of publicly funded health insurance (PFHI) for more than a decade now. PFHI in India is focused on in-patient care and it involves provision of services through public as well as private hospitals. In 2018, India increased the coverage of PFHI by introducing a new national scheme known as Pradhan Mantri Jan Arogaya Yojana (PMJAY). Demand side funding through cash incentives for users has also been implemented in India. Thus, there exist multiple mechanisms of financing for public and private hospitals in India now. An assessment of spending taking into account all its relevant components is needed across different types of providers in India. None of the existing studies have reported on different demand and supply side components of unit spending on hospitalisation. None of the existing studies on hospitalisation in India have examined the per-episode spending by public and private sources simultaneously. Nor have they attempted a comparison of the above spending for public and private hospitals. The current study is aimed at filling the above gaps in literature.

The objectives of the current study were the as following:

- to find out the relevant components of spending per episode of hospitalisation in terms of demand and supply side spending from public and private sources in one state of India
- to compare the total spending per episode of hospitalisation for public and private hospitals

## Materials and methods

### Study area

The study was carried out in Chhattisgarh state in India in 2019. The state had a population of around 29 million and 77% of it lived in rural areas [14]. A larger share of the state’s employment was in informal sector [15]. Earlier studies have reported that around 60% of the hospitalisations in Chhattisgarh took place in public hospitals [15]. Private hospitals in the state mostly belonged to for-profit category. The not-for-profit hospitals handled a very small share of hospitalisations in the state [15].

Around two-third of Chhattisgarh’s population was covered under PFHI schemes including PMJAY [15]. The PFHI schemes covered a wide-range of in-patient services. The PFHI schemes included ‘cash-less’ hospital-care under 1370 packages, covering all medical expenses [15, 16]. Both private and public hospitals were empanelled to provide services under PFHI. It was the main form of health insurance in the state. A negligible proportion of population was enrolled under private health insurance [14, 15].

Irrespective of the PFHI, public facilities were expected to provide healthcare at nominal charges except for a few tertiary-care procedures. User fees formed a very small part of the funding of public hospitals [17]. No user fees were applicable for the maternal and child healthcare [18]. Patients belonging to the below-poverty-line category were also exempt from user fees in public facilities. Public facilities provided most of the prescribed medicines free of cost to patients [19].

Cash incentives were paid by the government to women giving birth in institutions. All women delivering in public facilities were eligible to receive a cash incentive (INR 1400 for a rural case, INR 1000 for urban). In private facilities, women from the below-poverty-line category were eligible for the above incentive. Cash incentives were being paid by the government for sterilisation surgeries in public hospitals but the number of cases was quite small [20]. Apart from the users, cash incentives were paid to community health workers (CHWs) known as accredited social health activists (ASHA) for motivating women to go for institutional deliveries in public facilities (INR 300 per rural case, INR 200 per urban case) [18]. They received incentives for referrals for sterilization surgery, cataract surgery and hospitalisation of severely malnourished children but the numbers of cases involved were very small [20].

The government health facilities in Chhattisgarh like most states of India were organized in different tiers - a sub health centre (SHC) at 3000 to 5000 population providing preventive and maternal-care services, a primary health centre (PHC) at 20,000 to 30,000 population for primary health care services including inpatient services, a community health centre (CHC) at 80,000 to 120,000 population with medical officers providing secondary care services including inpatient care, and a district hospital (DH) at around a million population providing secondary care services along with limited specialist care. In addition, there were medical college (MC) hospitals providing tertiary care services at around 5 million population each [21]. All these facilities handled broadly three kinds of functions: a) providing outpatient care b) providing inpatient care c) non-clinical public health functions. Additionally, medical college hospitals also handled medical education. The normative number of hospital-beds in SHC, PHC, CHC, DH and MC are 2, 10, 30, 100 and 500 respectively.

### Components of total spending on inpatient care

The approach in the current study consisted of finding the total spending by adding the expenditures by government (i.e. public spending) and households (i.e. private spending). This helped in ensuring that no expenditure got counted twice as public and private spending formed two mutually exclusive categories.

The components of total spending on inpatient care, according to sources of funding were [5]:

- Government spending:
- Supply-side public spending through line-item budgets on public health facilities
- Demand-side public spending through PFHI
- Public spending on cash incentives to users and promoters for specific kinds of hospital utilisation
- Private spending:
- OOPE borne by patients/families
- Spending through private insurance paid by individuals or employers
- Donations to not-for-profit non-government hospitals

The last two categories under private spending (categories v and vi above) were not included because they were not material in the context of Chhattisgarh. Private health insurance covered a very small proportion (less than 1 %) of the population in Chhattisgarh [14].The not-for-profit hospitals had a very small share in hospital utilisation in the state [15]. Also, overseas aid was not a significant component of healthcare funding.

Therefore, the relevant components of total spending for hospitalisation were:

i) Supply-side public spending on public facilities through line item budgets
ii) Demand-side public spending through PFHI
iii) Public spending on cash incentives for using and promoting certain services
iv) OOPE borne by patients

#### Data sources, sampling and data collection

This was a descriptive study and it relied on both facility-based and population-based data to find the average total spending per episode of hospitalisation. Two different datasets were used:

- Household survey: A large household survey on morbidity and utilisation was carried out in Chhattisgarh in November–December 2019. The survey used a structured questionnaire to collect data about hospitalisation of individuals in the previous 365 days, the type of provider utilised, self reported ailment for which hospitalisation took place, PFHI enrolment status, claim amount, cash incentives and OOPE. Due quality assurance measures were implemented in the survey. The list of ailments used in the survey was based on the list used by the National Sample Survey (NSS) in its survey on self reported morbidity and healthcare utilisation [22]. This survey was carried out by the State Health Resource Centre, a technical agency working for the Department of Health, Government of Chhattisgarh.
  The study used the above primary household survey although a secondary dataset with similar sample size was available on healthcare utilisation and OOPE. The available secondary dataset was of the 75th round of National Sample Survey (NSS). However, NSS did not provide information on many of the variables required for this study. NSS provided data on only one component of spending, namely the OOPE. It did not collect data on the amount of demand side public spending through PFHI. Neither did it include data on demand side spending by cash incentives to users. NSS did not include any survey of facilities which was necessary to find out supply side spending. For the purpose of the current study, it was important that data from households and facilities are collected around the same time. Therefore a primary survey was conducted.
  An additional benefit of conducting a fresh survey was related to the timing. The latest secondary dataset available was of 75th Round of NSS, in which data collection took place before July, 2018. A major health financing policy in the form of a national health insurance scheme called PMJAY was introduced in India including Chhattisgarh in September, 2018. The primary survey done for the current study collected data in 2019 end. It was able to cover the current situation of healthcare financing in Chhattisgarh.
  The survey had a representative sample of the state’s population. The key purpose of the household survey was to compare the OOPE and other demand side spending for hospital care in public and private hospitals. In order to meet this objective, the survey needed to cover enough number of hospitalisation episodes in public and private hospitals. The survey followed a two-stage sampling. For a Type-1 error of 5% and power of 95%, a sample size of 384 hospitalisations was required (with 40% share in private hospitals). This requirement was multiplied by 1.5 to account for the multi-stage sampling design. Thus there was a requirement of 576 hospitalisations in the sample. Based on earlier studies, a hospitalisation rate of around 4.1% was expected [14]. Thus around 14,000 individuals were to be covered to yield at least 576 hospitalisations. The survey was able to cover 3000 households with 15,470 individuals. It covered 908 episodes of hospitalisation with around 40% of them occurring in private hospitals.
  Multi-stage sampling was followed. The state has five administrative divisions with a total of 27 districts. From each of the five geographical divisions in the state, one district was selected randomly. In each district, the number of rural and urban sampling units was decided in proportion to their share in population and selected through systematic random sampling.
  The household survey collected data on the monthly household consumption expenditure. The monthly household consumption expenditure of each household was divided by the number of family members to find out the per capita household consumption expenditure (MPCE). MPCE was calculated to create economic quintiles of the sample.
  The sample profile of individuals covered in the population-based survey is given in Additional File S1. It closely resembled the demographic profile for Chhattisgarh state available from existing sources such as the national census and NSS (75th round) (Additional File S1).
  b) Facility survey: A survey of public health facilities was carried out in January–February 2020 to find the supply-side expenditure per episode of hospitalisation. It collected data on various kinds of supply side spending, number of hospitalisations and the time allocation by various kinds of staff for inpatient care.
  For this facility survey, a sample of facilities was selected out of the public facilities in areas covered in the above mentioned household survey. This sample was also representative for the state. From each of the five districts covered in the household survey; one MC (where available), one DH, three CHCs, four PHCs and five SHCs were selected for the facility survey through systematic random sampling. The survey was able to cover 64 public facilities - 1MC, 5 DHs, 15 CHCs, 19 PHCs and 24 SHCs.

#### Data analysis

The following steps were carried out in data analysis:

- Finding out each of the relevant components of spending:
- Supply-side public spending on public facilities per hospitalisation: This was the government expenditure on inputs in public facilities for providing inpatient care, funded through supply-side budget. In the public facilities of the state, the account books of expenditure from supply-side budget were maintained separately from other sources of funds like user fees or insurance claims.

The expenditure on supply-side budget was calculated using the following data and methods:

- In the facility survey, data was collected from the accounts-officers regarding the annual recurring expenditure done by facility by using the supply-side budgets during January–December 2019.
- There were drugs and supplies received by facilities in kind from the state headquarters and expenditure on them was funded by the supply-side line-item budget. Data on the quantities of various types of drugs and supplies utilized (during January–December 2019) after being received in kind were collected from the facility stock-books and their valuation was carried out using price lists available from the state department.
- Costs of capital items like land, building and equipment were annualized. Cost of land was taken at current open market prices. Land was discounted at 7% per annum, considering the common market cost of borrowing capital in India was around 7%. Expenditure on buildings was discounted at 11% and equipments at 27%. Similar methods have been used to annualize capital costs in earlier studies in India [11–13].

The above expenditures were classified into five major heads:

- Human resources: staff salaries and training
- Infrastructure (annualised capital expenditure): land, building, equipments
- Materials and supplies: medicines, diagnostic consumables and supplies, other consumables
- Utilities: laundry, inpatient meals, electricity, telephone bills, stationery, contingency and housekeeping, ambulance running cost, travel, outreach camps
- Maintenance: building maintenance, equipment maintenance

In public facilities, most of the staff and infrastructure were deployed for multiple functions like outpatient care, inpatient care and non-clinical activities. Counting the expenditure directly for inpatient care alone was not possible in this context. An apportioning strategy was applied to attribute the costs to inpatient care. The total of the above expenditures was apportioned to inpatient care based on the proportion of working time spent by the facility’s staff on inpatient care and related services.

The staff from each cadre were interviewed to find the average proportion of time spent by that cadre on their three kinds of functions: outpatient care, inpatient care and the non-clinical activities. As done in other studies, the above information on time use was collected for one week period [4]. The proportion of time spent by each cadre on inpatient care was weighted with the share of that cadre in total salary bill of sampled facilities of a type. The above proportions were added for all the cadres to get the combined share of time on inpatient care for a facility-type. This method of apportioning the total cost of a hospital to a specific function (e.g. inpatient care) has been used by some studies in India [4, 11]. Human resources constitute the biggest part of expenditure in health facilities in India [4, 11].

The per-episode average expenditure was computed by dividing the total annual government expenditure on inpatient care by the annual number of hospitalisation episodes.

ii) Demand-side public spending through PFHI per episode of hospitalisation: This was the government expenditure through the PFHI schemes i.e. the amount paid to hospitals in form of claims for hospitalisations under PFHI. This data was available episode-wise from the household survey. In Chhattisgarh, private and public facilities were empanelled under PFHI and the same benefits package and prices were applicable for both. The average spending per episode was calculated over the total episodes.
  The public hospitals kept the accounts of expenditures done under the line item budgets separately from the PFHI claims. The uses made of PFHI claims by public hospitals were also quite distinct from the operational expenditures done from the line budgets. There was no chance of double counting of operational costs.
  The PFHI claim once paid by the government got accounted as expenditure and hospitals do not forego any unspent balance. This was different than the case of line item budgets where hospitals forego any unspent balance out of the budgeted amount at the end of financial year. Therefore while detailed item wise expenditure had to be counted for spending out of line budgets, the entire PFHI claim amount for a hospitalisation was counted as demand side spending.
iii) Cash incentives for users and promoters: Episode-wise data on the cash incentives was available from the household survey. The average spending per episode was calculated over the total episodes.
iv) OOPE per episode of hospitalisation: OOPE was taken as the direct expenses incurred by the individual/family during the episode. It included: a) Medical expenses: paying the hospital bills, buying drugs or tests; b) Non-medical expenses: food and transport of patient and attendants [23]. Any cash payments received by the patient were subtracted to calculate the OOPE [23]. Data on OOPE was obtained from the household survey.
b. Calculating the total spending per episode for public hospitals and private hospitals: The above components of spending were added to find out the total cost per episode for both categories of hospitals.
c. Comparing the case-mix for public and private hospitals: The proportion of each ailment in the total volume of hospitalisations handled by public and private hospitals was compared. This was done in order to examine if there were any key differences in the case-mix handled by the two types of facilities. The data on case-mix handled by public and private facilities was obtained from the household survey. Case-mix here refers to the type of ailments or conditions for which hospitalisations took place.
d. Multivariable analysis to confirm the difference in spending in public and private hospitals: This statistical analysis was aimed at examining whether the expenditure differed significantly for public and private providers when other relevant variables including the case-mix and duration of hospitalisation were controlled.

A multivariable ordinary least squares (OLS) regression model was applied with logarithmic (log) transformation of OOPE as dependent variable to find out the association between OOPE and type of provider. The log transformation of OOPE was used to address the possibility of extreme values or skew in OOPE data. A multivariable quantile regression model was also applied for comparison as this method can address the skewed distribution of OOPE [24, 25]. Quantile regression was conducted for the median as well as for the 20th, 40th, 60th and 80th centiles.

A propensity-score matching (PSM) model was applied to confirm whether OOPE differed significantly between the episodes in public and private hospitals while other relevant characteristics of the episodes were matched. PSM has been recommended as a suitable method to compare different kinds of health facilities or providers because it can help in achieving a better balance in patient characteristics, especially the case-mix [26, 27].

The application of the PSM model includes computing the propensity scores and treatment effect. The average treatment effect on the treated (ATET) gets computed by taking the average of the difference between the observed and potential outcomes for each subject [28]. In the current analysis, the type of hospital utilised (public/private) was used as the treatment variable while applying the PSM model in STATA.

The independent variables for the model were selected based on existing studies in Chhattisgarh and India [9, 14, 16]. The variables included in the models were: per-capita household consumption expenditure quintiles, gender, education, individual’s enrolment status under PFHI, duration of hospitalisation, ailments and the type of provider utilised (public/private). The ailments included in the model were those differing significantly between the public and private hospitals in terms of their proportion in hospitalisations.

The above adjusted models were first applied with the variable on type of provider in two categories - private facilities and public facilities. In order to allow a comparison of private hospitals against different sized public facilities, the regressions were repeated after dividing the public facilities into three categories and reported in additional files. The categories were - small facilities (with not more than 10 beds i.e. SHC and PHC), midsize facilities (with 11–50 beds i.e. CHC) and large facilities (with more than 50 beds i.e. DH and MC).

For determinants of demand-side public spending through PFHI, the analysis involved two stages. In the first stage, a logistic regression model was applied to find out the determinants of a PFHI claim getting generated for inpatient episodes. In the second stage, a multivariable OLS model was applied to find out determinants of the size of claim amount. The logarithmic transformation of amount of claim was used in the above OLS model. For robustness, PSM models were applied, corresponding to the above two questions.

No regression was needed for the supply side public spending as it applied only to public facilities. Regression for cash incentives was abandoned because they formed a very small part of overall spending and applied mainly for using public facilities.

Significance was taken at 95% confidence (*p* < 0.05). All analysis was carried out using STATA 15 software.

## Results

### Type of providers used

The proportion of hospitalisation episodes handled by different providers is given in Table 1.

**Table 1:** Inpatient episodes handled by different types of providers

Type of Providers	Share in the utilisation of inpatient care (%) (***N*** = 903 episodes)
*Public Providers:*	
Sub Health Centre (SHC)	2.9
Primary Health Centre (PHC)	13.4
Community Health Centre (CHC)	16.1
District Hospital (DH)	17.5
Medical College Hospital (MC)	10.6
**All Public Providers**	**60.4**
**For-profit Private Hospitals**	39.6

Public facilities had a bigger share of inpatient episodes than the private hospitals (Table 1). Among the public facilities, DHs handled a bigger share of hospitalisations, followed by CHCs and PHCs. In private providers, for-profit hospitals accounted for most of the utilisation. The private not-for-profit providers accounted for five episodes of hospitalisation and they were excluded from the analysis.

The case-mix i.e. the ailments handled by the public and private providers are given in Additional File S2. It showed that the case-mix was largely similar between public and private hospitals. Deliveries formed a relatively greater share of case-mix in public hospitals whereas injuries, typhoid and menstrual problems contributed to a greater share of utilisation in the private sector.

#### Supply-side public spending on public facilities per hospitalisation

The per episode government expenditure on inputs in public facilities for providing inpatient care is given in Table 2.

**Table 2:** Supply-side public spending in public facilities (in INR)

Main heads of spending	Sub-Health Centre	Primary Health Centre	Community Health Centre	District Hospital	Medical Colleges
Human resources	421,200	3,027,620	17,077,128	51,730,560	310,383,360
Infrastructure (annualised capital expenditure)	451,969	1,106,357	7,564,400	9,003,000	54,018,000
Materials and supplies	112,046	1,301,813	5,294,848	15,805,214	94,831,284
Utilities	42,000	348,058	5,099,974	13,630,038	81,780,228
Maintenance	48,787	189,740	719,642	2,260,800	13,564,800
**Total**	**1,076,002**	**5,973,588**	**35,755,992**	**92,429,612**	**554,577,672**
Share of inpatient care in time-use of staff	6.5%	14.5%	36.4%	50.8%	54.4%
Annual number of episodes of inpatient care	27	183	1996	8179	36,601
Government cost per episode of inpatient care (INR)	2590	4733	6521	5741	8250

Table 2 shows that expenditure on human resources (HR) was the dominant part of supply-side spending, followed by infrastructure. The supply-side government spending per hospitalisation was greatest for medical college hospitals.

In order to estimate the overall average spending for all public facilities put together, a weighted average of spending in SHC, PHC, CHC, DH and MC was calculated with their respective share in hospitalisations as the weight. The supply-side public spending for public facilities overall was thus estimated as INR 6011 per episode of hospitalisation. According to the household survey, the average duration of a hospitalisation episode in public facilities was 4.6 days, thus giving an average supply-side spending per day of INR 1307.

#### Demand side public spending through PFHI per episode of hospitalisation

The claim amount earned by hospitals from the PFHI scheme, averaged over total hospitalisations by facility-type is given in Table 3.

**Table 3:** Government expenditure as PFHI claim amount in different public and private facilities (INR)

Type of provider	Total No. of Hospitalisations (A)	No. of Hospitalisations in which a claim was raised under PFHI (B)	Total claim amount under PFHI (INR) (C)	Average demand-side public spending per episode (INR) (D=C/A)
**All Providers**	903	305	3,322,137	3679
*Public Facilities:*				
SHC	26	0	0	**0**
PHC	120	22	137,470	**1146**
CHC	144	39	335,078	**2327**
DH	158	46	421,198	**2666**
Medical College	95	53	520,667	**5481**
**All Public facilities**	**543**	**160**	**1,414,413**	**2605**
**For-profit Private Hospitals**	**360**	**145**	**1,907,724**	**5299**

Table 3 shows that the average per-episode demand-side public spending through PFHI was greater in case of private hospitals than the public facilities. Public facilities were able to raise a claim under PFHI for 29% of the hospitalisation episodes handled by them whereas the proportion was 40% for the private hospitals. The logistic regression for occurrence of PFHI claim showed that inpatient episodes in private hospitals were more likely to result in a PFHI claim than public hospitals (adjusted odds ratio = 1.45, *p* = 0.02) (Table 4).

**Table 4:** Logistic regression for occurrence of PFHI claim

PFHI Claim generated (Yes/No)	Coefficient	SD	***P*** Value	95% CI	
**Per Capita Household Consumption Expenditure Quintile**					
Poorest	1				
Poor	1.68	0.38	0.02	1.08	2.62
Middle	1.35	0.32	0.20	0.85	2.15
Rich	1.37	0.32	0.18	0.87	2.18
Richest	0.69	0.18	0.15	0.42	1.14
**Education**					
Uneducated	1				
Primary	1.09	0.20	0.64	0.76	1.56
Secondary	0.91	0.23	0.70	0.55	1.49
Graduation and above	1.43	0.33	0.12	0.91	2.26
**Sex**					
Male	1				
Female	0.81	0.14	0.22	0.58	1.14
**Type of Provider**					
Public	1				
Private	1.45	0.11	0.02	1.07	1.98
**Duration of hospitalization**	1.05	0.01	< 0.001	1.02	1.08
**Disease**					
Typhoid	1.32	0.49	0.46	0.63	2.75
Delivery	1.17	0.24	0.45	0.78	1.75
Menstrual problem	1.51	0.93	0.51	0.45	5.03
Animal or Insect Bite	1.00				
Injury	1.41	0.40	0.24	0.80	2.47

*N* = 887

Pseudo R2 = 0.045

The OLS regression for logarithmic transformation of PFHI claim amount showed that the size of a claim was likely to be greater for episodes in private hospitals than public facilities (coefficient of 0.27 with *p* < 0.001) (Table 5). The OLS regression for log of PFHI claim amount when repeated after dividing the public facilities into size based categories showed that the PFHI claim amount of an episode in private facilities was significantly greater than the large public facilities (coefficient of 0.28 with *p* < 0.001) (Additional File S3).

**Table 5:** OLS regression for Log of PFHI claim

Log of Claim Amount	Coefficient	p value	95% CI	
**Per Capita Household Consumption Expenditure Quintile**				
Poorest	Reference			
Poor	0.12	0.28	−0.09	0.32
Middle	0.27	0.02	0.05	0.50
Rich	0.02	0.87	−0.20	0.23
Richest	0.14	0.28	−0.11	0.38
**Education**				
Uneducated	Reference			
Primary	−0.06	0.48	−0.23	0.11
Secondary	0.12	0.33	−0.12	0.35
Graduation and above	−0.16	0.13	−0.37	0.05
**Sex**				
Male	Reference			
Female	−0.04	0.62	−0.20	0.12
**Type of Provider**				
Public	Reference			
Private	0.27	< 0.001	0.41	0.13
**Duration of hospitalization**	0.04	< 0.001	0.03	0.05
**Typhoid**	−0.23	0.18	−0.57	0.11
**Menstrual problem**	0.80	< 0.001	0.60	0.99
**Animal or Insect Bite**	−0.31	0.24	−0.84	0.21
**Injury**	1.08	< 0.001	0.84	1.31

No. of Observations = 294

R-squared = 0.47

The PSM model for occurrence of PFHI claim showed that inpatient episodes in the private hospitals were more likely to involve generation of PFHI claims than public hospitals (coefficient of 0.07 with *p* < 0.05) (Additional File S4). The PSM model for log of PFHI claim amount showed that the size of claim was likely to be greater for episodes in private hospitals than public hospitals (coefficient of 0.41 with *p* < 0.001) (Additional File S4).

#### Cash incentives

The cash incentives paid by the government are given in Table 6. The average was calculated over the total number of hospitalisations in the facility.

**Table 6:** Spending on cash incentives (INR)

Type of provider	Total No. of Hospitalisations (A)	No. of Hospitalisation cases received a cash incentive (E)	Total incentive amount paid (INR) (F)	Average incentive amount per episode (INR) (G = F/A)
**All Providers**	903	173	233,200	**258**
*Public Facilities:*				
SHC	26	22	35,200	1354
PHC	120	41	62,600	522
CHC	144	38	48,200	335
DH	158	30	38,400	243
Medical College	95	17	19,800	208
**All Public facilities**	543	148	204,200	**376**
**For-profit Private Hospitals**	360	25	29,000	**81**

#### Average OOPE per hospitalisation

Table 7 provides the mean OOPE per hospitalisation, in different types of public and private facilities.

**Table 7:** Mean OOPE in different types of public and private facilities (INR)

Type of provider	Mean OOPE (INR)
**All Providers**	**15,477**
*Public Providers:*	
SHC	149
PHC	1254
CHC	1713
DH	5264
MC	10,170
**All public facilities**	**4041**
**For-profit Private Hospitals**	**32,632**

The average amount of OOPE per hospitalisation in private hospitals was INR 32632, as compared to OOPE of INR 4041 in public facilities. Medical OOPE formed 88.8% of OOPE in public facilities and 95.7% of OOPE in private facilities.

The OLS regression for log of OOPE showed that OOPE in private hospitals was significantly greater than public hospitals (coefficient of 2.91 with *p* < 0.001) (Table 8).

**Table 8:** Linear Regression for Log transformation of OOPE

Log of OOPE	Coefficient	p value	95% Confidence Interval	
**Per Capita Household Consumption Expenditure Quintile**				
Poorest	Reference			
Poor	0.01	1.00	−0.52	0.52
Middle	0.18	0.51	−0.36	0.72
Rich	0.37	0.18	−0.17	0.90
Richest	0.48	0.08	−0.06	1.01
**Education**				
Uneducated	Reference			
Primary	0.24	0.26	−0.18	0.65
Secondary	0.41	0.15	−0.15	0.97
Graduation and above	1.01	< 0.001	0.48	1.55
**Sex**				
Male	Reference			
Female	−0.07	0.72	−0.47	0.32
**Type of Provider**				
Public	Reference			
Private	2.91	< 0.001	3.27	2.55
**Duration of hospitalization**	0.09	< 0.001	0.06	0.12
**PFHI enrolment**				
Yes	Reference			
No	−0.17	0.52	−0.68	0.34
**Typhoid**	−0.22	0.63	−1.11	0.67
**Menstrual problem**	−2.25	< 0.001	−2.71	−1.79
**Delivery**	0.25	0.74	−1.22	1.72
**Injury**	0.76	0.03	0.07	1.45

OLS Model:

No. of Observations = 887

R-squared = 0.40

The OLS regression for log of OOPE when repeated after dividing the public facilities into size based categories showed that the OOPE of an episode in private facilities was significantly greater than the large public facilities (coefficient of 2.21 with *p* < 0.001) (Additional File S5).

The quantile regressions for OOPE were applied for the median, 20th, 40th, 60th and 80th centiles. All the above quantile regressions showed that an episode in private hospitals was significantly more expensive than public hospitals. All the above adjusted models also showed that there was no significant association between OOPE and enrolment under PFHI. The results of the quantile regression have been provided in Additional File S6. The quantile regression when repeated after dividing the public facilities into size based categories showed that the OOPE of an episode in private facilities was significantly greater than the large public facilities (Additional File S6**)**.

The PSM model for log of OOPE with type of hospital used as treatment variable showed that the private hospitals were more expensive than public hospitals (coefficient of 2.04 with *p* < 0.001). The PSM model output is available in Additional File S4.

#### Total spending per hospitalisation

Table 9 gives the total spending per episode for utilisation by each type of facilities. It combines the findings of Tables 2, 3, 6 and 7.

**Table 9:** Total spending (in INR) per episode of hospitalisation in public and private providers by type of financing and its share (in %)

Type of provider	Supply side public spending (INR)	Demand side public spending through PFHI (INR)	Public spending on cash incentives (INR)	OOPE (INR)	Total spending (INR)
**All Hospitals**	**3609 (16%)**	**3679 (16%)**	**258 (1%)**	**15,477 (67%)**	**23,023 (100%)**
*Public Providers:*					
SHC	2590	0	1354	149	4093
PHC	4733	1146	522	1254	7655
CHC	6521	2327	335	1713	10,896
DH	5741	2666	243	5264	13,914
MC	8250	5481	208	10,170	24,109
**All public facilities**	**6011 (46%)**	**2605 (20%)**	**376 (3%)**	**4041 (31%)**	**13,033 (100%)**
**For-profit Private Hospitals**	**0 (0%)**	**5299 (14%)**	**81 (0.2%)**	**32,632 (86%)**	**38,012 (100%)**

Overall, cash incentives formed a very small component of the total spending. OOPE constituted the biggest component of total spending at 67%. The per hospitalisation amount of demand-side public spending was similar to the supply-side spending, each contributing to 16% of total spending. OOPE constituted 86% of spending per episode in private hospitals and 31% for public hospitals.

The total spending per episode in private hospitals was around three times greater than public facilities overall.

#### Total spending per day of hospitalisation

Table 10 gives the total spending per day of hospitalisation in each facility-type.

**Table 10:** Average total spending per day of hospitalisation (INR)

	Total Spending per episode of hospitalisation (INR)	Mean duration of an episode of hospitalisation (days)	Average spending per day of hospitalisation (INR)
**All Facilities**	23,023	5	**4605**
*Public Providers:*			
SHC	4093	1.8	2274
PHC	7655	2.9	2640
CHC	10,896	4.5	2421
DH	13,914	5.5	2530
MC	24,109	6.2	3889
**All Public Facilities**	13,033	4.6	**2833**
**For-profit Private Hospitals**	38,012	5.6	**6788**

The average per-day expenditure of hospitalisation in private hospitals was 2.4 times greater than the public facilities.

## Discussion

Existing studies in India have reported the supply-side expenditure for hospitalisation in CHCs and DHs. A recent study has reported the mean government expenditure of hospitalisation per episode as INR 2502 for DHs and INR 1601 for CHCs [3]. The supply-side government expenditure per hospitalisation in the current study in Chhattisgarh was greater than the above estimates. The difference can be partially explained by the difference in volume handled by the public facilities. For example, the average volume handled by a district hospital in the above study was around three times the volume in current study and it got reflected in the average expenditure per episode. A large share of operational cost of hospitals tends to be fixed in nature and therefore a similar sized facility handling a greater volume of patients is likely to have a lower average cost per episode [11]. Another recent study found the per day supply-side expenditure for in-patient care in public facilities (CHC) in India as INR 866 [4]. This amount when adjusted for inflation is around 20 % lower than the finding in current study, though the volume of patients handled was similar. The above difference seems to be due to the greater cost of human resources reported in the current study.

Few studies have covered the demand side public spending through PFHI in India. The current study found that this was a significant component of spending on inpatient care in Chhattisgarh and its size was similar to the supply side public spending per hospitalisation. A greater proportion of hospitalisations in the private sector resulted in a claim being raised under PFHI, as compared to public hospitals. Reports based on the existing programme data on PMJAY have shown that the private sector received a bigger share of total claim amount than the public sector [29].

The current study found that the amount of OOPE per hospitalisation in private hospitals was several times greater than the public hospitals. An adjusted model for determinants of OOPE while controlling the case-mix, duration of hospitalisation and other relevant variables showed that an episode in a private hospital was likely to cost substantially more than a public hospital. Existing studies from Chhattisgarh and other parts of India have reported similar findings [8–10, 14, 15, 30, 31].

The most important finding of the current study was in terms of the comparison of total spending per episode of hospitalisation for private and public providers. It covered both the spending by government as well as the households. This represents the average expenditure incurred by society for an episode of hospitalisation. Earlier studies have limited their comparison of spending for public and private providers to OOPE alone [8]. It has been argued that comparison based on OOPE alone could be unfair to the private sector as public facilities receive considerable subsidies from the government [32]. The current study took into account all relevant parts of spending on in-patient care and found that the for-profit private hospitals were several times more expensive than public facilities.

Why were the private hospitals more expensive? Many studies have reported that over-charging from patients remains a common practice in the Indian private sector [33–37]. Another factor could be the tendency of private providers to push unnecessary and costly procedures, drugs and tests [38–45]. Many studies from India have reported this phenomenon [10, 36, 46–52]. India has poor regulation of private providers in terms of prices and quality [36, 46, 52]. Medicines are one of the important contributors to healthcare costs in India and the use of bulk-purchased generic drugs could have helped the public sector in keeping their expenditure low [19].

What lessons emerge for improving financing for inpatient care in contexts similar to Chhattisgarh? The current study found that OOPE constituted 67% of the total spending. To make progress towards UHC, the financial burden on households needs to be reduced. Implementation of PFHI has been advocated as a remedy for controlling OOPE [53]. Yet, OOPE in the private hospitals was found to be very high. The adjusted model for determinants of OOPE showed that enrolment under PFHI was not associated with a reduction in OOPE. Most studies on PFHI in India have found it to be ineffective in reducing OOPE [15, 30–33, 54, 55]. PFHI in India has relied heavily on private hospitals in a situation of poor regulation. The benefit stipulated in PFHI was of free cashless services and contracts forbade hospitals from charging any copayments from patients [15, 30]. However, studies have reported widespread practice of ‘double-billing’ under PFHI where private hospitals claim the amount from insurance side while also charging illegal copayments from patients [56, 57]. Studies have shown that increasing the annual sum covered per family under PFHI could not improve its effectiveness in controlling OOPE [15, 30]. The current study covered the early days of the PMJAY policy but it seems that the key limitations of earlier PFHI schemes have persisted in its implementation. A qualitative study has highlighted the failure of contracts in regulating provider behavior and the normative and cultural context in which over-charging persists under PFHI in India [37]. Considering the above findings, demand-side spending through PFHI does not seem to be an effective strategy for reducing OOPE in the Indian context. It suggests that government should redirect these resources to increase supply-side spending.

India’s public expenditure on healthcare does not compare well with other LMICs like Sri Lanka and Thailand which have achieved greater progress on UHC [58, 59]. India’s per-capita public expenditure on healthcare was one-third of Sri Lanka’s and one-eighth of Thailand’s in 2018 in Purchasing Power Parity (PPP) terms [60]. India’s national health policy (2017) has recommended an increase in public spending on healthcare from 1% of Gross Domestic Product to 2.5% [61]. It is abundantly clear that the current per capita public expenditure on healthcare in India needs to be increased for attaining UHC. But how well the public expenditure is directed is also important. The current study suggests the likelihood that private hospitals charged way above their cost of production. This can have a bearing on the allocative efficiency in the health system [62]. Using the scarce public resources for purchasing inpatient care services from private providers may not be a suitable strategy for such contexts. The findings of this study have implications for the appropriate provider mix for the health system. The current study suggests that an increase in the share of public hospitals in utilisation can bring down overall expenditure on hospital care in the state. To increase their share in utilisation, public hospitals will need to attract more patients by offering better services. This may require an increase in supply-side spending as public facilities remain under-funded in India [10].

This is the first study that has presented a comprehensive picture of spending per episode of hospitalisation in an Indian state by including both public and private expenditures. To our knowledge, none of the studies in any part of India have reported the total spending per episode of hospitalisation. The current study provides a fair comparison of total spending per hospitalisation in public and private hospitals. Another advantage of the present study is that it was able to cover the period after introduction of the PMJAY policy.

In terms of methods, the current study took into account the demand as well as the supply side spending. It was aimed at finding the total spending per episode of hospitalisation and it did so by covering the relevant components of spending comprehensively. The study offers a feasible approach to compare average spending for different kinds of healthcare and providers. The approach may be useful for India as well as other countries, especially the LMICs. Depending upon the context, the relevant types of supply- and demand-side expenditures can be identified and included. The findings would differ according to the context but such comparisons can help in drawing lessons to inform the healthcare financing policies.

The current study also adds to the sparse literature available on comparisons between different kinds of healthcare providers in India. Further studies are recommended to carry out such comparisons in LMIC contexts while controlling for quality of care and health outcomes. Further research is also recommended to find the implications of such comparisons on financing strategies for UHC.

### Limitations

We acknowledge the possibility of recall bias in self-reporting in the household survey. Data on number of beds and specialties was not collected for private hospitals and therefore their effect on spending could not be analysed. The severity and complexity of illness can affect spending but it could not be captured. Some studies have reported that private hospitals tend to refer the more complicated cases to tertiary public hospitals [49, 63, 64]. Quality of care is an important variable but the current study could not include it. Quality of care is poorly regulated in private hospitals in India [46]. There has been little conclusive evidence in India whether private hospitals provided a better quality of care than public hospitals [10, 32, 37–40, 63]. It has been suggested that greater competition among providers can influence cost but the current study did not examine this aspect [62]. Comparison of average spending with the not-for-profit private hospitals can shed further light on the issue being examined but the study could not provide any findings on it. The study could not cover the hospitals owned by industrial enterprises.

## Conclusions

The current study provides a fair comparison of total spending per episode for public and private hospitals in the Indian state of Chhattisgarh. Government and households put together spent three times more on an episode of hospitalisation in a private hospital than a public facility. This finding has important implications for the allocative efficiency and the desired public-private provider-mix. Using public resources for purchasing inpatient care services from private providers may not be a suitable strategy for such contexts. For making progress towards UHC, further research is needed that compares spending for care in public and private sectors while linking it to their health outcomes.

## Supplementary Information

**Additional file 1.**

**Additional file 2.**

**Additional file 3.**

**Additional file 4.**

**Additional file 5.**

**Additional file 6.**

## Data Availability

The datasets used and/or analyzed during the current study are available from the corresponding author and State Health Resource Centre, Chhattisgarh on reasonable request.

## Abbreviations

ASHA: Accredited Social Health Activist
CHC: Community Health Centre
CHW: Community Health Worker
DH: District Hospital
INR: Indian Rupee
LMIC: Low and Medium Income Countries
MC: Medical College Hospital
OOPE: Out-of-pocket expenditure
PFHI: Publicly Funded Health Insurance
PHC: Primary Health Centre
SHC: Sub Health Centre

## References

[1] World Health Organization (2010). Health systems financing: The path to universal coverage. World Health Report.

[2] Barber SL, Lorenzoni L, Ong P. Price Setting and Price Regulation in Health Care. Price Setting and Price Regulation in Health Care: World Health Organization; 2019. https://apps.who.int/iris/handle/10665/325547

[3] Bahuguna P, Guinness L, Sharma S, Chauhan AS, Downey L, Prinja S (2020). Estimating the unit costs of healthcare service delivery in India: addressing information gaps for Price setting and health technology assessment. Appl health econ health policy.

[4] Prinja S, Chauhan AS, Bahuguna P, Selvaraj S, Muraleedharan VR, Sundararaman T (2020). Cost of delivering secondary healthcare through the public sector in India. PharmacoEconomics - open.

[5] National Health Systems Resource Centre. National Health Accounts Estimates for India: 2016–17. New Delhi; 2019. Accessed 6 Apr 2021

[6] Patel V, Parikh R, Nandraj S, Balasubramaniam P, Narayan K, Paul VK, Kumar AKS, Chatterjee M, Reddy KS (2015). Assuring health coverage for all in India. Lancet..

[7] Key Indicators of Social Consumption in India: Health. NSS 75th round. July 2017–June 2018.Govenrment of India, ministry of statistics and Programme implementation. National Statistics Office http://mospi.nic.in/sites/default/files/publication_reports/KI_Health_75th_Final.pdf.

[8] Pandey A, Clarke L, Dandona L, Ploubidis GB (2018). Inequity in out-of-pocket payments for hospitalisation in India: evidence from the National Sample Surveys, 1995-2014. Soc Sci Med.

[9] Hooda SK. Growth of Formal and Informal Private Healthcare Providers in India: Structural Changes and Implications. J Health Care Finance. 2017;44(2).

[10] Chatterjee S, Levin C, Laxminarayan R. Unit Cost of Medical Services at Different Hospitals in India. PLoS ONE. 2013;8(7). 10.1371/journal.pone.0069728 PMC3720595 23936088

[11] Prinja S, Balasubramanian D, Jeet G, Verma R, Kumar D (2017). Cost of delivering secondary-level health care services through public sector district hospitals in India. Indian J Med Res.

[12] Prinja S, Gupta A, Verma R, Bahuguna P, Kumar D (2016). Cost of delivering health Care Services in Public Sector Primary and Community Health Centres in North India. PLoS ONE.

[13] Nandi S, Schneider H, Dixit P (2017). Hospital utilisation and out of pocket expenditure in public and private sectors under the universal government health insurance scheme in Chhattisgarh state, India: lessons for universal health coverage. PLoS ONE.

[14] Garg S, Bebarta KK, Tripathi N. Performance of India’s national publicly funded health insurance scheme, Pradhan Mantri Jan Arogaya Yojana (PMJAY), in improving access and financial protection for hospital care: Findings from household surveys in Chhattisgarh state. BMC Public Health. 2020;20. 10.1186/s12889-020-09107-4 PMC7298746 32546221

[15] Government of India. About PradhanMantri Jan ArogyaYojana (PMJAY) https://www.pmjay.gov.in/about-pmjay. Accessed 6 Apr 2021 .

[16] Planning Commission of India. High Level Expert Group Report on Universal Health Coverage for India. Working Papers. 2011. Available from: http://ideas.repec.org/p/ess/wpaper/id4646.html%5Cnhttp://planningcommission.nic.in/reports/genrep/rep_uhc0812.pdf. Accessed 6 Apr 2021.

[17] Tripathi N, Kerketta F, Chatterjee P, Raman VR, John D, Jain K (2018). Access and availability of essential medicines in Chhattisgarh: situation in public health facilities. J Family Med Prim Care.

[18] National Health Systems Resource Centre (2019). Update on ASHA Programme.

[19] Chokshi M, Patil B, Khanna R, Neogi SB, Sharma J, Paul VK, Zodpey S (2016). Health systems in India. J Perinatol.

[20] Government of India (2016). Health in India – Health. NSS 71st Round. January 2014-June 2014. Ministry of Statistics and Programme Implementation. National Sample Survey Office.

[21] Kastor A, Mohanty SK (2018). Disease-specific out-of-pocket and catastrophic health expenditure on hospitalization in India: Do Indian households face distress health financing. PLoS ONE.

[22] Zhao Y, Atun R, Anindya K, McPake B, Marthias T, Pan T, Heusden A, Zhang P, Duolikun N, Lee JT (2021). Medical costs and out-of-pocket expenditures associated with multimorbidity in China: quantile regression analysis. BMJ Glob Health.

[23] Ali MS, Prieto-Alhambra D, Lopes LC, Ramos D, Bispo N, Ichihara MY, et al. Propensity Score Methods in Health Technology Assessment: Principles, Extended Applications, and Recent Advances. Front Pharmacol. 2019;10. 10.3389/fphar.2019.00973. 10.3389/fphar.2019.00973 PMC6760465 31619986

[24] Press STATA. STATA treatment effects reference manual: potential outcomes/counterfactual outcomes. Texas; 2015.

[25] Dong D, Sehgal P, Chhabra S, Naib P, Smith O (2020). PM-JAY: The Role of Private Hospitals. PM-JAY Policy Brief 9.

[26] Garg S, Chowdhury S, Sundararaman T (2019). Utilisation and financial protection for hospital care under publicly funded health insurance in three states in southern India. BMC Health Serv Res.

[27] Ranjan A, Dixit P, Mukhopadhyay I, Thiagarajan S (2018). Effectiveness of government strategies for financial protection against costs of hospitalisation Care in India. BMC Public Health.

[28] Olalere N (2020). Pause and reflect.

[29] Gadre A (2015). India’s private healthcare sector treats patients as revenue generators. BMJ..

[30] Nandi S, Schneider H (2020). Using an equity-based framework for evaluating publicly funded health insurance programmes as an instrument of UHC in Chhattisgarh state, India. Health Res Policy Sys.

[31] Maurya D, Ramesh M (2019). Program design, implementation and performance: the case of social health insurance in India. Health Econ Policy Law.

[32] Hooda SK, Private Sector in Healthcare Delivery Market in India: Structure, Growth and Implications. ISID Working Paper 185 (2015). Institute for Studies in industrial development.

[33] Nandi S, Schneider H (2019). When state-funded health insurance schemes fail to provide financial protection: an in-depth exploration of the experiences of patients from urban slums of Chhattisgarh. India Glob Public Health.

[34] Basu S, Andrews J, Kishore S, Panjabi R, Stuckler D (2012). Comparative performance of private and public healthcare systems in low- and middle-income countries: a systematic review. PLoS Med.

[35] UNDP (2015). Is the private sector more efficient? A cautionary tale. UNDP Global Centre for Public Service Excellence.

[36] Mackintosh M, Channon A, Karan A, Selvaraj S, Zhao H, Cavagnero E (2016). What is the private sector? Understanding private provision in the health systems of low-income and middle-income. Lancet..

[37] Seyedin H, Afshari M, Isfahani P, Rakhshan A, Hasanzadeh E, Taherimirghaed M (2020). Main Factors Leading to Supplier-Induced Demand in Iran: A Comprehensive Review. Health Scope.

[38] Stuckler D, Feigl A, Basu S, McKee M. The political economy of universal health coverage. Background paper for the global symposium on health systems research. Technical report. 2010. WHO; Geneva.

[39] Prince R (2017). Universal health coverage in the global south: new models of healthcare and their implications for citizenship, solidarity and the public good. Tidsskriftet Michael.

[40] Thresia CU (2013). Rising private sector and falling ‘good health at low cost’: health challenges in China, Sri Lanka, and Indian state of Kerala. Int J Health Serv.

[41] Hanson K, Gilson L, Goodman C, Mills A, Smith R, Feachem R, et al. Is private health care the answer to the health problems of the world’s poor? PLoS Med. 5(11):e233. 10.1371/journal.pmed.0050233. 10.1371/journal.pmed.0050233 PMC2586358 19067483

[42] Bhat R (1999). Characteristics of private medical practice in India: a provider perspective. Health Policy Plan.

[43] Sengupta A, Mukhopadhyaya I, Weerasinghe MC, Karki A (2017). The rise of private medicine in South Asia. BMJ..

[44] Deolalikar AB, Jamison DT, Jha P, Laxminarayan R (2008). Financing Health Improvements in India. Health Aff.

[45] Dasgupta R, Nandi S, Kanungo K, Nundy M, Murugan G, Neog R (2013). What the good doctor said: a critical examination of design issues of the RSBY through provider perspectives in Chhattisgarh, India. Social Change.

[46] Purohit BC (2001). Private initiatives and policy options: recent health system experience in India. Health Policy Plan.

[47] Garg P, Nagpal J (2014). A Review of Literature to Understand the Complexity of Equity, Ethics and Management for Achieving Public Health Goals in India. J Clin Diagn Res.

[48] Mills A (2014). Health Care Systems in low- and Middle-Income Countries. N Engl J Med.

[49] Preker AS, Lindner ME, Chernichovsky D, Schellekens OP (2013). Scaling up affordable health insurance.

[50] Prinja S, Bahuguna P, Gupta I, Chowdhury S, Trivedi M (2019). Role of insurance in determining utilisation of healthcare and financial risk protection in India. PLoS ONE.

[51] Karan A, Yip W, Mahal A (2017). Extending health insurance to the poor in India: an impact evaluation of Rashtriya Swasthya Bima Yojana on out of pocket spending for healthcare. Soc Sci Med.

[52] Devadasan N, Seshadri T, Trivedi M, Criel B (2013). Promoting universal financial protection: evidence from the Rashtriya Swasthya Bima Yojana (RSBY) in Gujarat. India Heal Res Policy Syst.

[53] Rent P And Ghosh S. Understanding the cash-less nature of government sponsored health insurance schemes: evidence from Rajiv Gandhi Jeevandayee Aarogya Yojana in Mumbai. SAGE Open.2015: 1–10.

[54] Smith O, Sri Lanka: achieving pro-poor universal health coverage without health financing reforms (2018). universal health coverage study series no. 38.

[55] Limwattananon S, Neelsen S, O’Donnell O, Prakongsai P, Tangcharoensathien V, van Doorslaer E (2015). Universal coverage with supply-side reform: the impact on medical expenditure risk and utilisation in Thailand. J Public Econ.

[56] World Health Organization (WHO). Global Spending on Health: A World in Transition 2019. Global Report. 2019; Available from: https://www.who.int/health_financing/documents/health-expenditure-report-2019/en/.

[57] National Health Policy (2017). Ministry of Health and Family Welfare, Government of India.

[58] Baru RV (2013). Challenges for regulating the private health services in India for achieving universal health care. Indian J Public Health.

[59] Lahariya C (2020). Stronger government health sub-system is the way to advance universal health coverage in India. J Med Evid.

[60] Muraleedharan VR, Vaidyanathan G, Sundararaman T, Dash U, Ranjan A, Rajesh M. Invest More in Public Healthcare Facilities What Do NSSO 71st and 75th Rounds Say. Econ Polit Wkly. 2020;lV(37).

[61] Government of India, Ministry of Health and Family Welfare, Maternal Health Division; 2011. National rural health mission. Guidelines for Janani-Shishu Suraksha Karyakram (JSSK) pp 1–40 Available from:https://nhmgovin/images/pdf/programmes/guidelines-for-jsskpdf Accessed 19 Apr 2021.

[62] Anindya K, Ng N, Atun R, Marthias T, Zhao Y, McPake B, van Heusden A, Pan T, Lee JT (2021). Effect of multimorbidity on utilisation and out-of-pocket expenditure in Indonesia: quantile regression analysis. BMC Health Serv Res.

[63] Littnerova S, Jarkovsky J, Parenica J, Pavlik T, Spinar J, Dusek L. Why to use propensity score in observational studies? Case study based on data from the Czech clinical database AHEAD 2006–09. Cor et Vasa. 2013;55(4).

[64] Bel G, Esteve M (2020). Is private production of hospital services cheaper than public production? A Meta-regression of public versus private costs and efficiency for hospitals. Int Public Manag J.

