# Supplementary material for: How much do government and households spend on an episode of hospitalisation in India? A comparison for public and private hospitals in Chhattisgarh state
Source: Health Econ Rev. 2022 May 6;12:27. doi: 10.1186/s13561-022-00372-0 (PMC9078002; doi:10.1186/s13561-022-00372-0)
Supplement: Supplementary file 1 — Additional file 1. [file 13561_2022_372_MOESM1_ESM.docx]

**Additional File S1: Sample Profile**

**Table S1.1- Household characteristics and Demography of sampled Household Members and Inpatients in Chhattisgarh (in 2019)**

| **Household characteristics and Demography of Household Members** | | **All Individuals (N=15470)** | **Inpatients (N=903)** |
| --- | --- | --- | --- |
| Place (Urban/Rural) | Urban | 3305(21.4%) | 231 (25.6%) |
|  | Rural | 12165 (78.6%) | 672 (74.4%) |
| Household size | Mean | 5.88 | 5.56 |
|  | Median | 6 (5-6) | 5 (5-5) |
| Occupation | Formal Sector | 1646 (10.6%) | 116 (12.8%) |
|  | Self-Employed | 7807 (50.5%) | 468 (51.8%) |
|  | Informal Sector | 5842 (37.8%) | 303 (33.5%) |
|  | Unemployed | 61 (0.4%) | 7 (0.8%) |
|  | Others | 114 (0.7%) | 9 (1.0%) |
| Social Group (Caste) | Scheduled Tribes (ST) | 5204 (33.6%) | 283 (31.4%) |
|  | Scheduled Castes (SC) | 1818 (11.8%) | 122 (13.5%) |
|  | Other Backward Classes (OBC) | 7928 (51.2%) | 474 (52.5%) |
|  | Others | 520 (3.4%) | 24 (2.7%) |
| Per-capita Household Consumption Expenditure in Quintiles | Q5 (Richest) | 2834(18.3%) | 188 (21.7%) |
|  | Q4 (Rich) | 3054 (19.7%) | 165 (19.%) |
|  | Q3 (Middle) | 2941(19%) | 164 (18.9%) |
|  | Q2 (Poor) | 2766 (17.9%) | 147 (16.9%) |
|  | Q1 (Poorest) | 3158(20.4%) | 204 (23.5%) |
| Sex of Individual | Male | 7681 (49.7%) | 334 (37%) |
|  | Female | 7789 (50.3%) | 569 (63%) |
| Age Category of Individual | <5 years | 1319 (8.5%) | 64 (7.1%) |
|  | 5-14 Years | 2823 (18.2%) | 64 (7.1%) |
|  | 15-48 Years | 8767 (56.7%) | 581 (64.5%) |
|  | 49-59 Years | 1231 (8%) | 77 (8.5%) |
|  | > 60 Years | 1268 (8.2%) | 115 (12.8%) |
| Education of Individual | Not Literate | 4650 (30.1%) | 277 (30.8%) |
|  | Primary | 6764 (43.7%) | 340 (37.8%) |
|  | High school | 1801 (11.6%) | 127 (14.1%) |
|  | Graduation and above | 2222 (14.4%) | 156 (17.3%) |
| Individual’s Coverage with Insurance | PMJAY | 7037 (45.5%) | 432 (47.8%) |
|  | MSBY | 3382 (21.9%) | 187 (20.7%) |
|  | Other | 108 (0.7%) | 8 (0.9%) |
|  | No Insurance | 4943 (32%) | 276 (30.5%) |

**Comparison of sample profile with other surveys in Chhattisgarh**

**Table S1.2: Key demographic characteristics of population of Chhattisgarh in different surveys**

| **Variables** | **Categories** | **Current Study in Chhattisgarh** | **National Sample Survey (NSS 75^th^ Round) Chhattisgarh**** | **National Census Chhattisgarh***** |
| --- | --- | --- | --- | --- |
|  |  | **Year 2019** | **Year 2017-18** | **Year 2011** |
|  |  | **N=15470** | **N=15025** |  |
| Place | Urban | 20.7 | 18.8% | 23.2% |
|  | Rural | 79.3 | 81.2% | 77.8% |
| Caste | Scheduled Tribes (ST) | 33.6% | 31.2% | 30.6% |
|  | Scheduled Castes (SC) | 11.8% | 16.2% | 12.8% |
|  | Other Backward Classes (OBC) | 51.2% | 46.5% | * |
|  | Others | 3.4% | 6.2% | * |
| Gender | Male | 49.7% | 52.6% | 50.2% |
|  | Female | 50.3% | 47.4% | 49.8% |
| Age | < 5 years | 8.5% | 6.9% | 9.9% |
|  | 5-14 Years | 18.2% | 19.7% | 22.1% |
|  | 15-48 Years | 56.7% | 56.5% | 53.1% |
|  | 49-59 Years | 8.0% | 11.9% | 4.0% |
|  | > 60 Years | 8.2% | 4.9% | 11.0% |
| Education | Not Literate | 30.1% | 24.6% | 29.7% |
|  | Primary | 43.7% | 49.2% | * |
|  | High school | 11.6% | 20.1% | * |
|  | Graduation and above | 14.4% | 5.9% | * |

* not available

** Source: Authors’ analysis of National Sample Survey (NSS 75^th^ Round) Chhattisgarh dataset

***Source: Registrar General of India. Census 2011 - Population Enumeration Data. Available at https://censusindia.gov.in/2011census/population_enumeration.html
