# Supplementary material for: How much do government and households spend on an episode of hospitalisation in India? A comparison for public and private hospitals in Chhattisgarh state
Source: Health Econ Rev. 2022 May 6;12:27. doi: 10.1186/s13561-022-00372-0 (PMC9078002; doi:10.1186/s13561-022-00372-0)
Supplement: Supplementary file 2 — Additional file 2. [file 13561_2022_372_MOESM2_ESM.docx]

**Additional File S2: Case-Mix in Public and Private Hospitals**

**Table: Proportion of different ailments in inpatient care episodes managed by public and private providers**

| **Disease/Condition** | **Public (N=543)** | **Private (N=360)** | **p-value**  **(Chi-square)** |
| --- | --- | --- | --- |
| Delivery (Child birth) | 26.7% | 17.2% | <0.01 |
| Malaria | 8.1% | 9.4% | 0.47 |
| Diarrhea | 7.7% | 5.6% | 0.21 |
| Stomach ache | 6.5% | 5.0% | 0.36 |
| Injuries | 5.7% | 9.4% | 0.03 |
| Weakness | 5.2% | 5.8% | 0.68 |
| Typhoid | 3.4% | 6.4% | 0.04 |
| Body Ache | 3.2% | 2.2% | 0.37 |
| Cold and cough | 2.7% | 2.8% | 0.94 |
| Cataract | 2.5% | 2.5% | 0.99 |
| Sickle Cell Disease | 1.8% | 1.9% | 0.87 |
| Hypertension | 1.8% | 1.9% | 0.87 |
| Diabetes | 1.6% | 1.9% | 0.71 |
| Pneumonia | 1.6% | 1.4% | 0.79 |
| Antenatal care (ANC) | 1.6% | 0.6% | 0.15 |
| Tuberculosis | 1.4% | 1.7% | 0.78 |
| Cardiovascular Disease | 1.3% | 3.1% | 0.06 |
| Knee or Joint Pain | 1.1% | 0.8% | 0.72 |
| Jaundice | 0.9% | 1.1% | 0.75 |
| Stroke | 0.9% | 1.1% | 0.75 |
| Menstrual Problems | 0.7% | 2.5% | 0.03 |
| Eye Infection | 0.7% | 1.7% | 0.18 |
| Respiratory Infection | 0.7% | 0.3% | 0.38 |
| Poisoning | 0.7% | 0.3% | 0.38 |
| STI & Urinal Infection | 0.7% | 1.7% | 0.18 |
| Severe acute malnutrition | 0.0% | 0.6% | 0.97 |
| Mental Illness | 0.5% | 0.3% | 0.56 |
| Asthma | 0.5% | 0.8% | 0.59 |
| Skin Infection | 0.5% | 0.0% | 0.16 |
| Cancer | 0.4% | 0.8% | 0.34 |
| Sterilisation | 0.4% | 0.6% | 0.66 |
| Chicken Pox | 0.4% | 0.0% | 0.26 |
| Postpartum Complication | 0.4% | 0.0% | 0.26 |
| Burn | 0.0% | 0.3% | 0.75 |
| Ear Infection | 0.2% | 0.6% | 0.33 |
| Animal or Insect Bite | 0.0% | 0.0% | 0.42 |
| Others | 6.1% | 6.6% | 0.8 |
| Unknown | 0.5% | 1.1% | 0.33 |
| Total | 100.0% | 100.0% |  |
