# Supplementary material for: How much do government and households spend on an episode of hospitalisation in India? A comparison for public and private hospitals in Chhattisgarh state
Source: Health Econ Rev. 2022 May 6;12:27. doi: 10.1186/s13561-022-00372-0 (PMC9078002; doi:10.1186/s13561-022-00372-0)
Supplement: Supplementary file 3 — Additional file 3. [file 13561_2022_372_MOESM3_ESM.docx]

**Additional File S3: Adjusted Model for PFHI claim amount**

**Table: Linear (OLS) regression for Log of PFHI claim amount – with public facilities in three categories**

|  | | | |
| --- | --- | --- | --- |
| **No. of Observations=294** | **R-squared=0.47** |  |  |

| **Log of Claim Amount** | **Coefficient** | **p value** | **95% CI** | |
| --- | --- | --- | --- | --- |
| **Per Capita Household Expenditure Quintile** |  |  |  |  |
| Poorest | Ref. |  | | |
| Poor | 0.114 | 0.291 | -0.098 | 0.326 |
| Middle | 0.272 | 0.018 | 0.047 | 0.497 |
| Rich | 0.012 | 0.913 | -0.209 | 0.233 |
| Richest | 0.128 | 0.311 | -0.120 | 0.377 |
| **Education** |  |  |  |  |
| Uneducated | Ref. |  |  |  |
| Primary | -0.060 | 0.486 | -0.230 | 0.109 |
| Secondary | 0.119 | 0.322 | -0.117 | 0.354 |
| Graduation and above | -0.162 | 0.132 | -0.374 | 0.049 |
| **Sex** |  |  |  |  |
| Male | Ref. |  |  |  |
| Female | -0.043 | 0.602 | -0.204 | 0.118 |
| **Type of Provider** |  |  |  |  |
| Large public facility | Ref. |  |  |  |
| Small public facility | -0.011 | 0.933 | -0.269 | 0.247 |
| Midsize public facility | 0.045 | 0.712 | -0.195 | 0.285 |
| Private facility | 0.279 | <0.001 | 0.120 | 0.438 |
| **Duration of hospitalization** | 0.037 | <0.001 | 0.027 | 0.048 |
| Typhoid | -0.228 | 0.192 | -0.571 | 0.115 |
| Menstrual problem | 0.793 | <0.001 | 0.593 | 0.993 |
| Delivery | -0.317 | 0.241 | -0.849 | 0.215 |
| Injury | 1.074 | <0.001 | 0.835 | 1.312 |
