# Supplementary material for: How much do government and households spend on an episode of hospitalisation in India? A comparison for public and private hospitals in Chhattisgarh state
Source: Health Econ Rev. 2022 May 6;12:27. doi: 10.1186/s13561-022-00372-0 (PMC9078002; doi:10.1186/s13561-022-00372-0)
Supplement: Supplementary file 4 — Additional file 4. [file 13561_2022_372_MOESM4_ESM.docx]

**Additional File S4 – Propensity Score Matching Models for OOPE and PFHI claim**

**Table 4.1: Propensity Score Matching (PSM) Model for occurrence of PFHI Claim - with all diseases**

| Number of obs = 887 | |  |  |  |  |  |
| --- | --- | --- | --- | --- | --- | --- |
|  |  |  |  |  |  |  |
| **ATET (PFHI claim)** | **Coefficient** | **P Value** | **[95% Conf. Interval]** | |  |  |
| Private (with public as reference) | 0.07 | 0.04 | 0.01 | 0.14 |  |  |
|  |  |  |  |  |  |  |
|  |  |  |  |  |  |  |
|  |  |  |  |  |  |  |

**Table 4.2: Propensity Score Matching (PSM) Model for occurrence of PFHI Claim - with four diseases**

| Number of obs= 887 | |  |  |  |  |  |
| --- | --- | --- | --- | --- | --- | --- |
|  |  |  |  |  |  |  |
| **ATET (PFHI claim)** | **Coefficient** | **P Value** | **[95% Conf. Interval]** | |  |  |
| Private (with public as reference) | 0.09 | 0.02 | 0.01 | 0.16 |  |  |
|  |  |  |  |  |  |  |
|  |  |  |  |  |  |  |
|  |  |  |  |  |  |  |

**Table 4.3: Propensity Score Matching (PSM) Model for Log of PFHI Claim Amount with all diseases**

|  |  | Number of obs = 294 | | |  |  |
| --- | --- | --- | --- | --- | --- | --- |
|  |  |  |  |  |  |  |
| **ATET (Log of PFHI Claim Amount)** | **Coefficient** | **P Value** | **[95% Conf. Interval]** | |  |  |
| Private (with public as reference) | 0.407 | <0.001 | 0.215 | 0.598 |  |  |
|  |  |  |  |  |  |  |
|  |  |  |  |  |  |  |
|  |  |  |  |  |  |  |

**Table 4.4: Propensity Score Matching (PSM) Model for Log of PFHI Claim Amount - with four diseases**

|  | | | | | | |
| --- | --- | --- | --- | --- | --- | --- |
| Number of obs = 294 | |  |  |  |  |  |
|  |  |  |  |  |  |  |
| **ATET (Log of PFHI Claim Amount)** | **Coefficient** | **P Value** | **[95% Conf. Interval]** | |  |  |
| Private (with public as reference) | 0.291 | <0.001 | 0.077 | 0.506 |  |  |
|  |  |  |  |  |  |  |
|  |  |  |  |  |  |  |
|  |  |  |  |  |  |  |

**Table 4.5: Propensity Score Matching (PSM) Model for Log of OOPE – with all diseases**

| Number of obs = | 887 |  |  |  |  |  |
| --- | --- | --- | --- | --- | --- | --- |
|  |  |  |  |  |  |  |
| **ATET (Log of OOPE)** | **Coefficient** | **P Value** | **[95% Conf. Interval]** | |  |  |
| Private (with public as reference) | 2.043 | <0.001 | 1.682 | 2.404 |  |  |
|  |  |  |  |  |  |  |
|  |  |  |  |  |  |  |
|  |  |  |  |  |  |  |


|  | |  |  |  |  |  |
| --- | --- | --- | --- | --- | --- | --- |
|  |  |  |  |  |  |  |
|  |  |  |  | |  |  |
|  |  |  |  |  |  |  |
|  |  |  |  |  |  |  |
|  |  |  |  |  |  |  |
|  |  |  |  |  |  |  |

**Table 4.6: Propensity Score Matching (PSM) Model for Log of OOPE – with four diseases**

| Number of obs = 887 | |  |  |  |  |  |
| --- | --- | --- | --- | --- | --- | --- |
|  |  |  |  |  |  |  |
| **ATET (Log of OOPE)** | **Coefficient** | **P Value** | **[95% Conf. Interval]** | |  |  |
| Private (with public as reference) | 3.319 | <0.001 | 2.829 | 3.809 |  |  |
|  |  |  |  |  |  |  |
|  |  |  |  |  |  |  |
|  |  |  |  |  |  |  |

|  | | | | | | |
| --- | --- | --- | --- | --- | --- | --- |
|  | |  |  |  |  |  |
|  |  |  |  |  |  |  |
|  |  |  |  | |  |  |
|  |  |  |  |  |  |  |
|  |  |  |  |  |  |  |
|  |  |  |  |  |  |  |
|  |  |  |  |  |  |  |
