# Supplementary material for: How much do government and households spend on an episode of hospitalisation in India? A comparison for public and private hospitals in Chhattisgarh state
Source: Health Econ Rev. 2022 May 6;12:27. doi: 10.1186/s13561-022-00372-0 (PMC9078002; doi:10.1186/s13561-022-00372-0)
Supplement: Supplementary file 5 — Additional file 5. [file 13561_2022_372_MOESM5_ESM.docx]

**Additional File S5**

**Table: Linear (OLS) Regression for Log transformation of OOPE - with public facilities in 3 categories**

|  |  | | |  |  |  |  |  |
| --- | --- | --- | --- | --- | --- | --- | --- | --- |
|  | | | | | | | | |
|  |  |  |  |  |  |  |  |  |
| **No. of Observations=887** | **R-squared=0.44** | | | |  |  |  |  |
| **Log of OOPE** | | **Coefficient** | **P Value** | | | **95% CI** | |  |
| **Per Capita Household Expenditure Quintile** | |  |  | | |  |  |  |
| Poorest | | Ref. |  | | | | |  |
| Poor | | -0.20 | 0.45 | | | -0.70 | 0.31 |  |
| Middle | | -0.04 | 0.88 | | | -0.56 | 0.48 |  |
| Rich | | 0.10 | 0.72 | | | -0.43 | 0.62 |  |
| Richest | | 0.31 | 0.25 | | | -0.21 | 0.83 |  |
| **Education** | |  |  | | |  |  |  |
| Uneducated | | Ref. |  | | | | |  |
| Primary | | 0.20 | 0.32 | | | -0.20 | 0.61 |  |
| Secondary | | 0.20 | 0.47 | | | -0.34 | 0.75 |  |
| Graduation and above | | 0.91 | 0.00 | | | 0.39 | 1.43 |  |
| **Sex** | |  |  | | |  |  |  |
| Male | | Ref. |  | | | | |  |
| Female | | 0.03 | 0.88 | | | -0.35 | 0.41 |  |
| **Type of Provider** | |  |  | | |  |  |  |
| Large public facility | | Ref. |  | | | | |  |
| Small public facility | | -1.95 | <0.001 | | | -2.47 | -1.42 |  |
| Midsize public facility | | -1.23 | <0.001 | | | -1.76 | -0.70 |  |
| Private facility | | 2.21 | <0.001 | | | 1.81 | 2.62 |  |
| **Duration of hospitalization** | | 0.07 | <0.001 | | | 0.04 | 0.10 |  |
| **PFHI** | |  |  | | |  |  |  |
| Yes | | Ref. |  | | | | |  |
| No | | -0.21 | 0.40 | | | -0.71 | 0.28 |  |
| **Disease** | |  |  | | |  |  |  |
| Typhoid | | 0.11 | 0.80 | | | -0.75 | 0.98 |  |
| Menstrual problem | | -2.01 | <0.001 | | | -2.46 | -1.56 |  |
| Delivery | | 0.46 | 0.53 | | | -0.96 | 1.88 |  |
| Injury | | 0.69 | 0.04 | | | 0.03 | 1.36 |  |
